# Development of Subunit Vaccines That Provide High-Level Protection and Sterilizing Immunity against Acute Inhalational Melioidosis

**DOI:** 10.1128/IAI.00724-17

**Published:** 2017-12-19

**Authors:** Mary N. Burtnick, Teresa L. Shaffer, Brittany N. Ross, Laura A. Muruato, Elena Sbrana, David DeShazer, Alfredo G. Torres, Paul J. Brett

**Affiliations:** a Department of Microbiology and Immunology, University of South Alabama, Mobile, Alabama, USA; b Department of Microbiology and Immunology, University of Nevada, Reno School of Medicine, Reno, Nevada, USA; c Department of Microbiology and Immunology, University of Texas Medical Branch, Galveston, Texas, USA; d Department of Pathology, Autopsy Division, University of Texas Medical Branch, Galveston, Texas, USA; e Bacteriology Division, U.S. Army Medical Research Institute of Infectious Diseases, Frederick, Maryland, USA; University of California San Diego School of Medicine

**Keywords:** *Burkholderia pseudomallei*, Hcp1, capsule, glycoconjugate, immunity, inhalation, melioidosis, mouse, protection, vaccines

## Abstract

Burkholderia pseudomallei, the etiologic agent of melioidosis, causes severe disease in humans and animals. Diagnosis and treatment of melioidosis can be challenging, and no licensed vaccines currently exist. Several studies have shown that this pathogen expresses a variety of structurally conserved protective antigens that include cell surface polysaccharides and cell-associated and cell-secreted proteins. Based on those findings, such antigens have become important components of the subunit vaccine candidates that we are currently developing. In the present study, the 6-deoxyheptan capsular polysaccharide (CPS) from B. pseudomallei was purified, chemically activated, and covalently linked to recombinant CRM197 diphtheria toxin mutant (CRM197) to produce CPS-CRM197. Additionally, tandem nickel-cobalt affinity chromatography was used to prepare highly purified recombinant B. pseudomallei Hcp1 and TssM proteins. Immunization of C57BL/6 mice with CPS-CRM197 produced high-titer IgG and opsonizing antibody responses against the CPS component of the glycoconjugate, while immunization with Hcp1 and TssM produced high-titer IgG and robust gamma interferon-secreting T cell responses against the proteins. Extending upon these studies, we found that when mice were vaccinated with a combination of CPS-CRM197 and Hcp1, 100% of the mice survived a lethal inhalational challenge with B. pseudomallei. Remarkably, 70% of the survivors had no culturable bacteria in their lungs, livers, or spleens, indicating that the vaccine formulation had generated sterilizing immune responses. Collectively, these studies help to better establish surrogates of antigen-induced immunity against B. pseudomallei as well as provide valuable insights toward the development of a safe, affordable, and effective melioidosis vaccine.

## INTRODUCTION

Melioidosis is an emerging infectious disease that is being increasingly recognized in tropical regions around the world. While it is known to be endemic in at least 48 different countries in Southeast Asia, South Asia, the Middle East, Africa, Central America, and South America, current models predict that the disease is probably endemic in 34 additional countries where it is yet to be reported (1). Underrecognition of melioidosis is due, in part, to the fact that most cases occur in resource-poor countries with large rural populations and limited microbiological laboratory capabilities (2). Since the clinical presentations of melioidosis are diverse, ranging from skin abscesses to acute pneumonia and septicemia, diagnosis can be difficult. In 2015, the estimated total global burden of human melioidosis was ∼165,000 cases with ∼89,000 deaths, which is equivalent to the number of deaths attributed to measles and exceeds the levels of leptospirosis and dengue virus infection, underscoring the potential impact of the disease worldwide (1).

Burkholderia pseudomallei, the etiologic agent of melioidosis, is a facultative intracellular Gram-negative bacterium that can be isolated from environmental niches such as rice paddies, still or stagnant waters, and moist soils in areas where melioidosis is endemic. Humans can acquire B. pseudomallei infections through percutaneous inoculation via skin abrasions during occupational or recreational exposure, inhalation of bacteria in aerosolized dust or water, or ingestion of contaminated water (3, 4). Most natural infections occur in individuals with one or more risk factors, such as diabetes, alcoholism, chronic pulmonary disease, chronic renal disease, or thalassemia (5–8). At present, the association between route of infection and the clinical manifestations of melioidosis has not been clearly defined. Recent studies, however, have demonstrated a link between inhalation of aerosolized B. pseudomallei during severe weather events and pneumonia (9–12). Notably, over half of all melioidosis cases present as pneumonia, which can range from mild to severe disease (13).

In addition to being an important public health concern, B. pseudomallei is considered a potential biological weapon and is currently categorized as a tier 1 select agent by the U.S. Centers for Disease Control and Prevention (14, 15). In the event of an intentional release, it is believed that the most likely mode of dissemination would be via infectious aerosols that would lead to respiratory disease. Since B. pseudomallei is intrinsically resistant to many conventionally used antibiotics, treatment of melioidosis can be complicated. For culture-confirmed cases, the currently recommended antibiotic regimens are lengthy and typically involve a minimum of 2 weeks of intravenous therapy followed by up to 6 months of oral therapy (13). The ability of B. pseudomallei to persist inside host cells makes eradication of infections difficult, and even with appropriate chemotherapeutic intervention, relapse is possible (13). Furthermore, reinfection with a different B. pseudomallei strain can occur following successful treatment. At present, there are no human vaccines available for immunization against melioidosis. Because of these challenges, the development of medical countermeasures to combat melioidosis has become a priority in recent years (16).

An ideal melioidosis vaccine would be one that provides long-term protection against the most severe forms of the disease, namely, acute pneumonia and septicemia, and broad-spectrum protection against multiple B. pseudomallei strains. Several different live-attenuated vaccine strains as well as B. pseudomallei-derived outer membrane vesicles (OMVs) have been evaluated in preclinical studies and shown to confer significant protection in animal models of melioidosis (17–20). There are, however, important safety concerns associated with these types of vaccines. Numerous studies have also shown that B. pseudomallei expresses a variety of structurally conserved protective antigens. These antigens include cell surface polysaccharides (e.g., 6-deoxyheptan capsular polysaccharide [CPS] and lipopolysaccharide), cell-associated proteins (e.g., LolC, OmpA, OmpW, and FliC), and secreted proteins (e.g., BopA, BimA, FlgL, and MprA) (16, 21–29). Although these subunit vaccine candidates offer safety advantages over the use of live-attenuated strains and OMVs, none has been able to provide complete protection and sterilizing immunity when tested alone (16).

Since CPS is structurally conserved and expressed by all known virulent isolates of B. pseudomallei, it is an attractive antigen for vaccine development (26, 30). Supporting this, CPS-specific monoclonal antibodies (MAbs) have been used to passively immunize mice against lethal challenges of B. pseudomallei (21, 28). Recently, we showed that immunization of BALB/c mice with a CPS-cBSA (cationized bovine serum albumin) glycoconjugate resulted in high CPS-specific IgG titers that conferred significant protection against a B. pseudomallei challenge (26). In addition, we have shown that when immunized with a combination of CPS-cBSA and recombinant LolC, mice exhibit higher survival rates when challenged with a lethal intraperitoneal dose of B. pseudomallei compared to mice immunized with each component alone (26). Based upon these observations, we believe that a subunit vaccine formulation that stimulates both protective humoral and cellular immune responses can be developed to provide full protection against B. pseudomallei infections.

In the present study, we used a combination of molecular genetic, biochemical, and immunological approaches to evaluate the immunogenicity and protective capacity of a CPS-based glycoconjugate combined with either a hemolysin coregulated protein (Hcp1) or a deubiquitinase (TssM). Here, we demonstrate for the first time that subunit vaccine formulations containing these antigens provide C57BL/6 mice with high-level protection and sterilizing immunity against acute inhalational challenge with B. pseudomallei.

## RESULTS

### Synthesis of CPS-CRM197.

**FIG 1:**
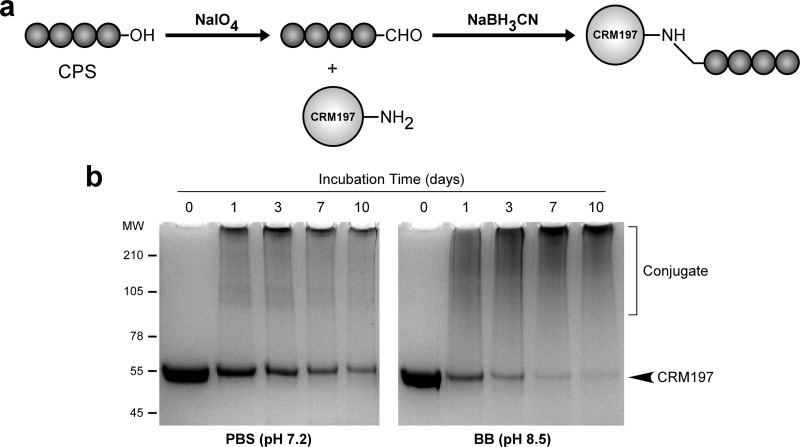
Synthesis and physical analysis of CPS-CRM197. (a) Basic conjugation strategy used to couple purified B. pseudomallei CPS to recombinant CRM197. (b) SDS-PAGE and Coomassie blue staining were used to assess the covalent linkage of CPS to CRM197 in PBS or BB. Samples were drawn from the reaction mixtures on days 0, 1, 3, 7, and 10. Day 0 represents unconjugated controls. All lanes were loaded with equal amounts of protein to facilitate direct comparisons. The positions of the protein molecular weight standards (molecular weights × 10^3^) are indicated on the left.

To construct the glycoconjugate material used in this study, the 6-deoxyheptan CPS from B. pseudomallei RR2683 (select agent excluded strain) was isolated by using a hot aqueous-phenol extraction method (30, 31). The purified CPS was then oxidized and covalently linked to CRM197 diphtheria toxin mutant (CRM197) via reductive amination to produce CPS-CRM197 (Fig. 1a). To optimize conjugation of the CPS to CRM197, small-scale reactions (with mixtures containing 5 mg CPS plus 2.5 mg CRM197) were initially conducted in phosphate-buffered saline (PBS; pH 7.2) or borate buffer (BB; pH 8.5). At various time points during the coupling reactions, samples were drawn and examined by SDS-PAGE (Fig. 1b). As indicated by shifts in molecular weights of the conjugate material relative to CRM197 controls, the results demonstrated that the CPS covalently linked to the carrier protein in a time-dependent manner. Reaction of the CPS with CRM197 was found to be most efficient in BB, with the majority of the carrier protein being coupled by day 10. Interestingly, while the conjugate material synthesized in BB was isolated in a soluble form, most of the conjugate material produced in PBS ended up as an insoluble precipitate. Based on these observations, a large-scale reaction of CPS (20 mg) with CRM197 (10 mg) was conducted using BB as the solvent system. Upon termination of the reaction, the yield of CPS-CRM197 was determined to be 23.3 mg (∼77% of the starting material). Analysis of the glycoconjugate material revealed that it contained 60% (wt/wt) CPS and 52 endotoxin units (EU)/mg as determined by protein and endotoxin assays, respectively. Similar to previous studies, Western immunoblotting confirmed that the structural integrity and antigenicity of the CPS remained intact following chemical activation and linkage to the carrier protein, based upon strong reactivity with MAb 3C5 (see Fig. S1 in the supplemental material) (30).

### Production of recombinant Hcp1 and TssM.

To obtain recombinant Hcp1 and TssM for use in the present study, N-terminal His-tagged versions of these proteins were expressed and purified from E. coli. Like our previous studies, the Hcp1 and TssM antigens were extracted from whole-cell pellets in a soluble form and purified to homogeneity using tandem nickel-cobalt chromatography (32–34). SDS-PAGE was used to assess the purity and structural integrity of the antigens (Fig. S2). Endotoxin concentrations associated with the Hcp1 and TssM preparations were 0.20 and 0.49 EU/mg, respectively, as determined in a Limulus amoebocyte lysate (LAL) assay.

### Analysis of antibody responses raised against CPS-CRM197, Hcp1, and TssM.

**FIG 2:**
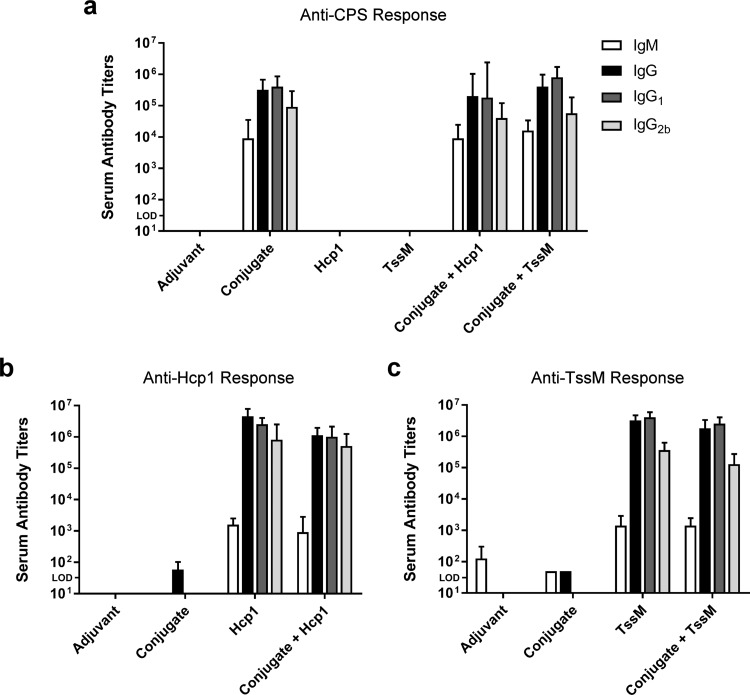
Characterization of antibody titers raised against CPS-CRM197, Hcp1, and TssM. C57BL/6 mice (*n* = 6 per group) were immunized on days 0, 21, and 35 with adjuvant only (Alhydrogel/CpG), conjugate only, Hcp1 only, TssM only, conjugate plus Hcp1, or conjugate plus TssM. Immune serum samples were collected for testing on day 42. ELISAs were used to quantitate serum IgM, IgG, IgG1, and IgG2b titers against (a) CPS, (b) Hcp1, and (c) TssM. Bars represent geometric means with 95% confidence intervals. “Conjugate” is CPS-CRM197; LOD, limit of detection.

To assess the immunogenic potential of our vaccine antigens, groups of C57BL/6 mice were immunized with (i) adjuvant only, (ii) CPS-CRM197, (iii) Hcp1, (iv) TssM, (v) CPS-CRM197 plus Hcp1, or (vi) CPS-CRM197 plus TssM. One week after the final boost, serum was collected from the mice, and antigen-specific IgM and IgG titers were determined in an enzyme-linked immunosorbent assay (ELISA). As expected, the conjugate (whether alone or in combination with Hcp1 or TssM) stimulated the production of a high-titer IgM response (endpoint titers of ≥10^3^) and a total IgG response (endpoint titers of ≥10^5^) against CPS (Fig. 2a). Similarly, the recombinant Burkholderia proteins (whether alone or in combination with CPS-CRM197) stimulated the production of high-titer IgM (endpoint titers of ≥10^3^) and total IgG (endpoint titers of ≥10^6^) responses against Hcp1 and TssM (Fig. 2b and c). Additionally, analysis of the immune serum samples revealed that balanced Th2/Th1-type responses were raised against all antigens based on the IgG1:IgG2b ratios (Fig. 2a to c) (35, 36).

**FIG 3:**
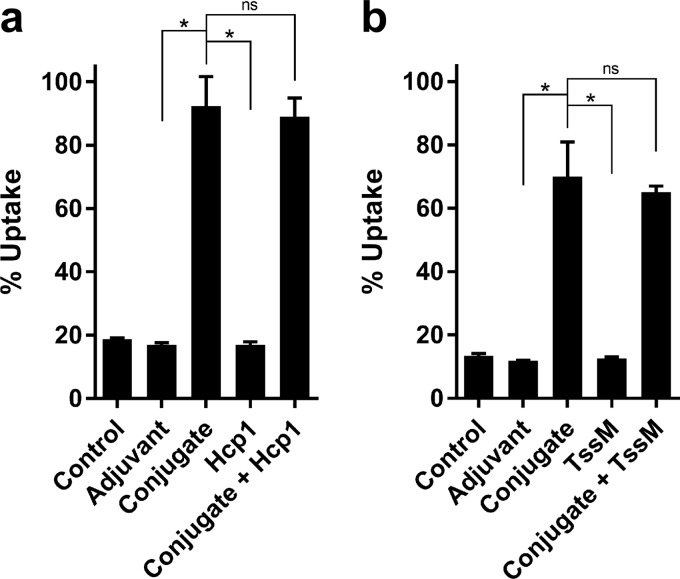
Functional analysis of antibody responses raised against CPS-CRM197, Hcp1, and TssM. C57BL/6 mice (*n* = 6 per group) were immunized on days 0, 21, and 35 with adjuvant only (Alhydrogel/CpG), conjugate only, Hcp1 only, TssM only, conjugate plus Hcp1, and conjugate plus TssM. Immune serum samples were collected on day 42. B. pseudomallei K96243 was incubated with (a) medium only (no-serum control), pooled HI adjuvant (Alhydrogel/CpG)-only immune serum, pooled HI conjugate-only immune serum, pooled HI Hcp1-only immune serum, pooled HI conjugate plus Hcp1-immune serum, or (b) medium only (no-serum control), pooled HI adjuvant (Alhydrogel/CpG)-only immune serum, pooled HI conjugate-only immune serum, pooled HI TssM-only immune serum, or pooled HI conjugate plus TssM immune serum. Following incubation for 1 h, opsonized bacteria were added to RAW 264.7 murine macrophage monolayers. Uptake was quantitated at 3 h postinfection. Reported values represent mean results ± standard deviations for three individual assays conducted in triplicate. Figures are representative of at least three independent experiments conducted on different days. ns, not significant; *, *P* < 0.05.

To assess the functionality of the CPS- and protein-specific antibodies, opsonophagocytosis assays were conducted. As shown in Fig. 3, preincubation of B. pseudomallei K96243 with heat-inactivated (HI) pooled antiserum from mice immunized with CPS-CRM197, CPS-CRM197 plus Hcp1, or CPS-CRM197 plus TssM significantly enhanced bacterial uptake into RAW 264.7 cells (>5-fold) compared to responses in mice administered nonconjugate antiserum controls. When serum obtained from groups of mice immunized with adjuvant only, Hcp1, or TssM was evaluated in the same assays, no differences in uptake levels were observed in comparison to the medium-only controls. Taken together, these results demonstrate that the conjugate material used in our vaccine formulations was capable of stimulating opsonizing antibody responses in C57BL/6 mice.

### Analysis of cellular immune responses against CPS-CRM197, Hcp1, and TssM.

**FIG 4:**
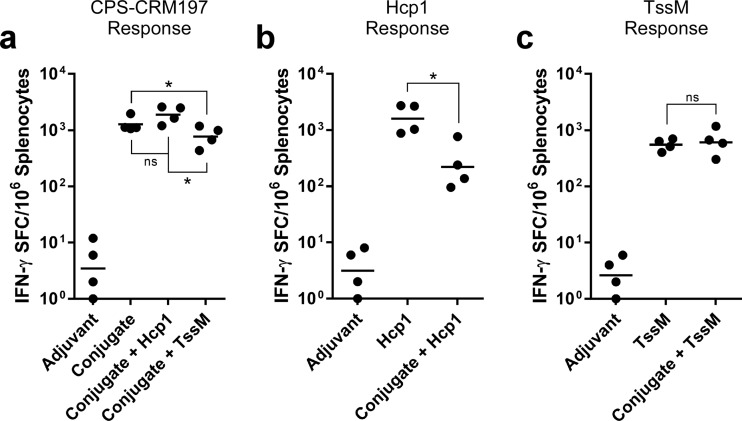
Characterization of cellular immune responses raised against CPS-CRM197, Hcp1, and TssM. C57BL/6 mice (*n* = 4 per group) were immunized on days 0, 21, and 35 with adjuvant only (Alhydrogel/CpG), conjugate only, Hcp1 only, TssM only, conjugate plus Hcp1, or conjugate plus TssM. Spleens were harvested on day 42, and IFN-γ-secreting T cell responses against (a) CPS-CRM197, (b) Hcp1, and (c) TssM were quantitated in an ELISpot assay. Black dots represent the mean results of assays conducted in duplicate for individual mice. Black bars represent geometric means for a group. ns, not significant; *, *P* < 0.05.

One week after the final boost, single-cell suspensions were prepared from the spleens of immunized mice and restimulated with the various vaccine antigens. Enzyme-linked immunosorbent spot (ELISpot) assays were then used to analyze T cell responses. Since CRM197 is a well-characterized T cell-dependent antigen, splenocytes isolated from mice immunized with CPS-CRM197, CPS-CRM197 plus Hcp1, or CPS-CRM197 plus TssM were restimulated with CPS-CRM197 for control purposes (37, 38). As shown in Fig. 4a, all conjugate-immunized mice exhibited robust gamma interferon (IFN-γ)-secreting T cell responses compared to responses of the mice that received adjuvant only. Similarly, splenocytes obtained from mice immunized with the Burkholderia proteins (whether alone or in combination with CPS-CRM197) also exhibited robust IFN-γ-secreting T cell responses when restimulated with Hcp1 or TssM, respectively (Fig. 4b and c). Interestingly, compared to one another, not all the immunized groups of mice displayed equivalent responses. For instance, when restimulated with conjugate material, splenocyte preparations from mice in the CPS-CRM197 plus TssM group had significantly lower numbers of IFN-γ-secreting T cells than mice immunized with CPS-CRM197 or CPS-CRM197 plus Hcp1 (Fig. 4a). Furthermore, when splenocytes from mice immunized with CPS-CRM197 plus Hcp1 were restimulated with Hcp1, fewer IFN-γ-secreting T cells were detected than in the mice immunized with Hcp1 only (Fig. 4b). No significant differences were observed, however, between the T cell responses obtained from mice immunized with either CPS-CRM197 plus TssM or TssM only when restimulated with TssM (Fig. 4c). Collectively, these findings demonstrate that robust IFN-γ-secreting T cell responses can be raised against the Burkholderia protein antigens used in this study.

### Animal challenge studies.

**FIG 5:**
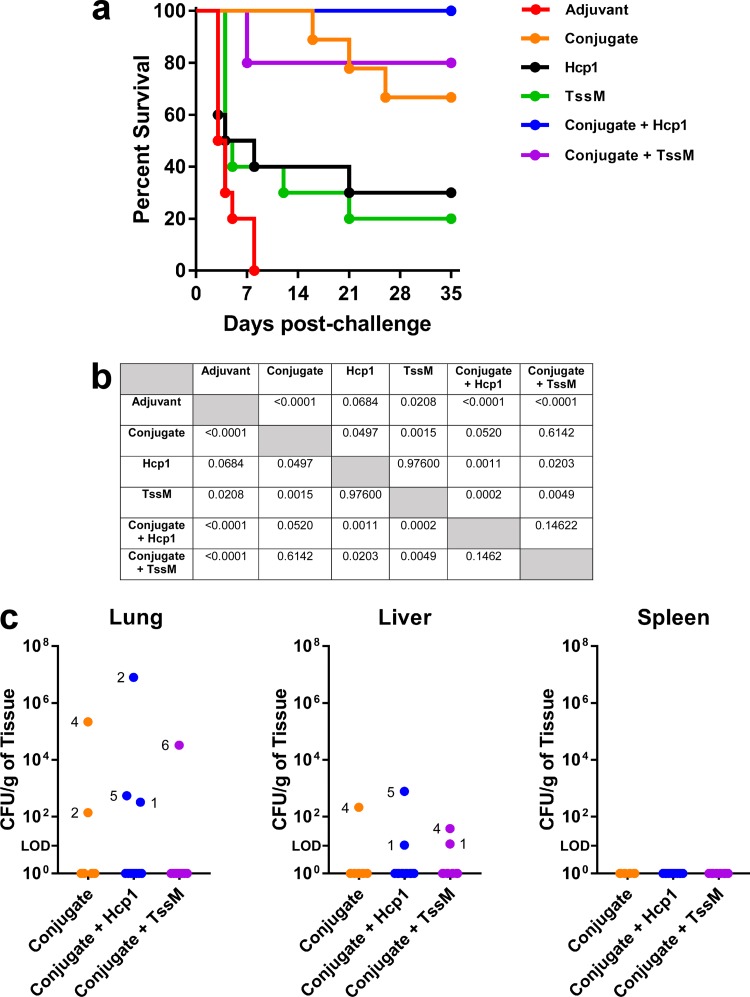
Protective capacities of the subunit vaccine formulations tested in this study. C57BL/6 mice (*n* = 9 to 10 mice per group) were immunized on days 0, 21, and 35 with adjuvant only (Alhydrogel/CpG), conjugate only, Hcp1 only, TssM only, conjugate plus Hcp1, or conjugate plus TssM. Five weeks after the final boost, mice were challenged via the inhalational route with ∼10 LD_50_ of B. pseudomallei K96243. (a) Mice were monitored for 35 days postchallenge, and their survival was plotted. (b) Significance for survival was determined using a log rank (Mantel-Cox) test. (c) At the end of the study, survivors were culled (*n* = 10 for conjugate plus Hcp1; *n* = 8 for conjugate plus TssM; *n* = 6 for conjugate only), organs were removed, and bacterial loads were determined. Individual mice were designated according to the numbers next to the colored dots.

The ultimate goal of this study was to assess the abilities of the various antigens to protect immunized mice against a lethal bacterial challenge. To facilitate this, we first had to establish a median 50% lethal dose (LD_50_) for our B. pseudomallei isolate, using appropriately aged mice. This was accomplished by challenging 16- to 18-week-old C57BL/6 mice with various doses of B. pseudomallei K96243 via an inhalational route and monitoring their survival for 35 days (Fig. S3). Upon termination of the experiment, we calculated the LD_50_ to be 154 CFU. Once this had been accomplished, immunized mice were challenged with ∼10 LD_50_ of K96243 5 weeks after the final boost and monitored for signs of morbidity and mortality over a 35-day period. As expected, all mice in the adjuvant-only group succumbed rapidly to infection (≤8 days). In contrast, groups immunized with CPS-CRM197 (whether alone or in combination with Hcp1 or TssM) exhibited survival rates that were significantly different from the adjuvant control (Fig. 5a and b). Specifically, immunization of mice with CPS-CRM197 plus Hcp1 resulted in 100% survival, while immunization with CPS-CRM197 plus TssM or CPS-CRM197 alone resulted in 80% and 67% survival, respectively. Interestingly, only 30% and 20% of the mice immunized with Hcp1 or TssM, respectively, survived the full duration of the study.

**FIG 6:**
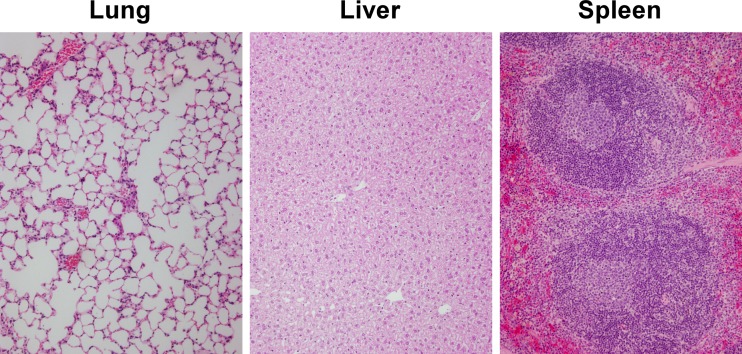
Histopathological analysis of mouse tissues following a lethal inhalational challenge with B. pseudomallei. Following termination of the challenge study, lungs, livers, and spleens were harvested from five mice (numbers 1 to 5) of the 10 survivors that had been immunized with CPS-CRM197 plus Hcp1. The tissues were fixed and stained with H&E. Images are representative of all 5 mice. Original magnification, ×400.

To further investigate the protective capacity of our vaccine formulations, survivors were culled at day 35, and tissue samples from lungs, livers, and spleens were plated to quantitate bacterial loads. As shown in Fig. 5c, 7/10 of the mice immunized with CPS-CRM197 plus Hcp1 had no culturable bacteria in any of their tissues. Interestingly, while mouse 2 from this group was shown to have high bacterial loads in its lungs, there was no evidence of dissemination to its liver or spleen. Results also demonstrated that 5/8 of the mice immunized with CPS-CRM197 plus TssM and 4/6 of the mice immunized with CPS-CRM197 had no detectable bacteria in their tissues. Remarkably, no bacteria were isolated from the spleens of any of the mice. Consistent with these findings, histopathological analyses revealed that the lungs, livers, and spleens from survivors in the group immunized with CPS-CRM197 plus Hcp1 (mice 1 to 5) were unremarkable and presented as normal healthy tissue with normal architecture (Fig. 6; Table S1). Taken together, these studies demonstrated the vaccinogenic potential of our antigen formulations. Additionally, they showed that immunization of C57BL/6 mice with CPS-CRM197 plus Hcp1 resulted in 100% survival and 70% sterilizing immunity following an acute inhalational challenge with B. pseudomallei.

## DISCUSSION

Melioidosis is now increasingly recognized as an important cause of morbidity and mortality worldwide, yet no licensed vaccines currently exist to prevent disease in humans or animals (1, 16). Since B. pseudomallei is a facultative intracellular pathogen, protective immunity is likely complex, requiring a combination of humoral and cell-mediated responses. To address this, studies in our laboratories have been focused on the development of subunit vaccines that incorporate a combination of protective polysaccharide and protein antigens (26, 29). The rationale for this approach is that when appropriately adjuvanted, such vaccines should be able to stimulate both protective antibody and T cell responses. Additionally, since these types of vaccines are antigenically defined, they can be designed to minimize the safety issues and undesirable side effects often associated with the use of whole-cell or live attenuated vaccines. In the present study, we provided strong evidence that our subunit vaccines can protect mice against an acute inhalational challenge with B. pseudomallei. Collectively, our results help to better establish surrogates of antigen-induced immunity against this important bacterial pathogen, as well as provide valuable insights toward the development of an antigenically defined, safe, affordable, and effective melioidosis vaccine (39).

The 6-deoxyheptan CPS is an attractive vaccine candidate because it is (i) a requisite virulence factor, (ii) structurally conserved, and (iii) expressed by all known virulent isolates of B. pseudomallei (40). Additionally, several passive and active immunization studies have proven it to be a protective antigen (21, 28). Notably, we previously showed that immunization of BALB/c mice with a CPS-cBSA conjugate provided significant protection against lethal intraperitoneal challenges with B. pseudomallei (26). While this earlier study was an important first step in demonstrating the vaccinogenic potential of CPS, we recognized that there were limitations regarding the use of animal proteins to develop human vaccines. In the present study, therefore, we wanted to identify a carrier that would facilitate efficient production of a CPS-based glycoconjugate but, unlike cBSA, could also be licensed for use in humans.

There are five different carrier proteins currently used to produce human glycoconjugate vaccines: tetanus toxoid (T), meningococcal outer membrane protein complex (OMPC), Haemophilus influenzae protein D (HiD), diphtheria toxoid (D), and CRM197 (41). Of these, CRM197 is considered the most versatile, since it has multiple lysyl side chains that are available for coupling with activated polysaccharides (41). Based on this, we elected to produce our conjugates using recombinant CRM197 (38). Initial attempts to conjugate CPS to CRM197 using PBS as a solvent system resulted in less-than-optimal conjugation and the formation of insoluble precipitates. To resolve these issues, we switched to a BB solvent system, which resulted in high-efficiency coupling and the production of soluble conjugate material that could be filter sterilized (42).

It is well established that capsular polysaccharides enable many bacterial pathogens to evade uptake and killing by phagocytic cells (43). It is also well established that CPS-based glycoconjugate vaccines can induce opsonizing antibody responses to combat disease caused by such organisms (44, 45). The intended purpose of the glycoconjugate material in our vaccine formulations, therefore, was to stimulate the production of opsonizing antibody responses against the CPS antigen. When immunized with CPS-CRM197, either alone or in combination with Hcp1 or TssM, C57BL/6 mice were found to generate high-titer CPS-specific IgG responses. Such findings are consistent with previous studies in which CPS-cBSA was used as an immunogen, and our findings confirmed that T cell-dependent-type antibody responses are being raised against the polysaccharide portion of CPS-CRM197 (46). Extending upon these findings, we also investigated the opsonizing capacity of the immune serum samples. As anticipated, our results demonstrated that CPS-specific antibodies, whether alone or in combination with Hcp1- or TssM-specific antibodies, were functional and promoted uptake of B. pseudomallei into RAW 264.7 cells. These findings are consistent with several previous studies demonstrating that CPS-specific monoclonal antibodies and/or polyclonal antiserum enhances uptake of B. pseudomallei and/or B. mallei into phagocytic cells (28, 30, 47, 48).

A variety of proteins have been explored for their vaccinogenic potential in animal models of melioidosis. Varied results have been achieved, with no single antigen providing high-level protection against acute challenges with B. pseudomallei (16). Recently, we demonstrated that when BALB/c mice were immunized with a combination of CPS-cBSA and recombinant LolC, rather than with CPS-cBSA or LolC individually, they exhibited higher survival rates when challenged with a lethal dose of B. pseudomallei (26). In the present study, we extended upon this finding by investigating the protective capacity of two different Burkholderia proteins combined with CPS-CRM197 in C57BL/6 mice. The proteins selected for this purpose were Hcp1 and TssM. Hcp1 is a major structural component of the virulence-associated B. pseudomallei type VI secretion system and is expressed at high levels upon activation of this system (32, 49). TssM is a potent deubitiquitinase involved in modulating host immune responses and is secreted by B. pseudomallei in a type II secretion system-dependent manner (34, 50). These proteins were chosen because (i) they are highly conserved among B. pseudomallei isolates, (ii) they are known to be expressed in humans during active infections with the organism, and (iii) Hcp1 is a known protective antigen in animal models of experimental melioidosis (32, 51). In addition to these important attributes, recombinant Hcp1 and TssM are both “well-behaved” proteins that can be expressed at high levels in Escherichia coli and purified in a soluble form, and they are stable for extended periods of time when stored at 4°C (data not shown). When immunized with recombinant Hcp1 or TssM, either alone or in combination with CPS-CRM197, C57BL/6 mice were found to produce high-titer IgG responses (endpoint titers of ≥10^6^) against the proteins. Such results indicated that Hcp1 and TssM are highly immunogenic antigens.

Since B. pseudomallei is capable of surviving and replicating within host cells, it is reasonable to predict that cell-mediated immune responses are important for controlling infections caused by this pathogen. For instance, B. pseudomallei protein-specific IFN-γ-secreting T cells are likely required to promote efficient clearance of the organism following uptake by macrophages. Supporting this, recent studies have demonstrated a correlation between survival of melioidosis patients and enhanced T cell immunity to specific B. pseudomallei antigens (52, 53). Thus, the intended purpose of the Burkholderia proteins in our vaccine formulations was to stimulate the production of Hcp1- and TssM-specific IFN-γ-secreting T cell responses. Results of ELISpot assays demonstrated that robust IFN-γ-secreting T cell responses were observed in both Hcp1- and TssM-immunized mice. Interestingly, when Hcp1 was combined with CPS-CRM197, the Hcp1-specific T cell responses were significantly lower than those in mice immunized with only Hcp1. At present, the reason for this phenomenon is unclear. Further studies are required to better understand this effect as well as determine whether it may influence vaccine efficacy. In addition to this, studies will be required to confirm which T cell populations (i.e., CD4^+^, CD8^+^, or both) are activated by the Burkholderia proteins, as well as what role(s) they might be playing in controlling B. pseudomallei infections.

Following three doses of the vaccine formulations, C57BL/6 mice were challenged with lethal inhalational doses (∼10 LD_50_) of B. pseudomallei K96243. This challenge route was chosen to evaluate the protective capacity of our vaccine antigens, since it represents a natural route of infection and would also be the most likely mode of exposure in the event of a deliberate release of the organism (54). When used alone, Hcp1 and CPS-CRM197 were shown to provide different degrees of protection (30% and 67% survival, respectively). Supporting our hypothesis that optimal protection likely requires both humoral and cellular immune responses, combining the antigens yielded an observable synergistic effect. Specifically, 100% of the mice immunized with CPS-CRM197 plus Hcp1 survived the full duration of the study. Although protection afforded by the antigen combination was not statistically different than that by CPS-CRM197 alone, there was a clear biological advantage associated with the use of this formulation. A major challenge in developing vaccines to combat disease caused by facultative intracellular pathogens is the host's ability to achieve sterilizing immunity (16, 55). Based upon the results of this study, there is compelling evidence that our lead vaccine formulation (CPS-CRM197 plus Hcp1) may be able to accomplish this goal. To our knowledge, this is the highest level of protection conferred by a subunit vaccine against an acute inhalational challenge with B. pseudomallei.

Collectively, these studies support our rationale for developing multivalent subunit vaccines to immunize against disease caused by B. pseudomallei. Considering that high-level protection was achieved against an acute inhalational challenge, we predict that such vaccines will be useful for both public health and biodefense purposes. Studies are ongoing to confirm our findings, optimize the protective capacity of our lead formulation, and define specific surrogates of antigen-induced immunity in both C57BL/6 (the prototypical Th1 strain) and BALB/c (the prototypical Th2 strain) mouse models of acute melioidosis.

## MATERIALS AND METHODS

### Bacterial strains, plasmids, and growth conditions.

Bacterial strains and plasmids used in this study are described in Table 1. Escherichia coli strains with plasmids were cultured on Luria-Bertani–Lennox (LB; Fisherbrand) agar or in LB broth containing ampicillin (100 μg/ml). B. pseudomallei RR2683 was cultured in LB broth or on LB agar supplemented with thiamine (5 μg/ml) and adenine (100 μg/ml). B. pseudomallei K96243 was cultured in LB broth or on LB agar. All bacterial cultures were incubated at 37°C; broth cultures were incubated with shaking (200 rpm). Bacterial stocks were maintained at −80°C as 20% glycerol suspensions. All manipulations of B. pseudomallei K96243 were conducted in CDC- and USDA-approved and registered biosafety level 3 (BSL3) or animal biosafety level 3 (ABSL3) facilities at the University of South Alabama or the University of Texas Medical Branch, and experiments were performed in compliance with the rules and regulations of the U.S. Federal Select Agent Program.

**TABLE 1:** Bacterial strains and plasmids used in this study

Strain or plasmid	Description^*a*^	Reference or source
Escherichia coli strain		
TOP10	Lab strain for cloning and protein expression	Life Technologies
Burkholderia pseudomallei strains		
RR2683	OPS-deficient derivative of select agent excluded strain Bp82; Δ*purM* Δ*rmlD*	61
K96243	Wild type; clinical isolate from Thailand	62
Plasmids		
pBAD/HisA	Arabinose inducible, 6× His tag expression vector; Ap^r^	Life Technologies
pBADBmhcp1-6HisF	pBAD/HisA containing *B. mallei hcp1* (BMAA0742) with an N-terminal His tag	33
pMB1000	pUC57Kan containing *B. pseudomallei tssM* (BPSS1512) corresponding to amino acids 191–474 in which Cys codon (TGC) at position 102 in TssM changed to Gly codon (GGC)	This study (GenScript)
pMB1001	pBAD/HisA containing *B. pseudomallei tssM* (BPSS1512) corresponding to amino acids 191–474 in which Cys codon (TGC) at position 102 in TssM changed to Gly codon (GGC); N-terminal His tag	This study

a Ap^r^, ampicillin resistance.

### CPS purification.

Broth in 2-liter baffled Erlenmeyer flasks was inoculated with B. pseudomallei RR2683 and incubated overnight at 37°C with shaking (200 rpm). Cell pellets were obtained by centrifugation and extracted using a modified hot aqueous phenol procedure (31). Purified CPS antigens were then obtained essentially as previously described (30, 56).

### Glycoconjugate synthesis.

Recombinant, preclinical-grade CRM197 was purchased from Reagent Proteins. The CPS-CRM197 glycoconjugates used in this study were synthesized essentially as previously described (30, 56). Briefly, purified CPS was solubilized at 5 mg/ml in PBS (BupH; Pierce) and added to a small amber vial. To each milliliter of the CPS solution was added ∼6 mg (∼30 mM) of sodium *meta*-periodate. Once the crystals had dissolved, the reaction mixture was incubated for ∼40 min at room temperature with stirring. To remove any excess oxidizing agent, the reaction mixture was applied to a Zeba Desalt spin column (Pierce) equilibrated with either PBS or BB (Pierce), and the eluate was collected. To facilitate conjugation of CPS to the carrier protein (CRM197 buffer exchanged at 5 mg/ml into either PBS or BB on a Zeba column), the activated CPS was added to a small amber vial. To each milliliter of the CPS solution was added 500 μl of the carrier protein (5-mg/ml stock). Following mixing by gentle agitation, 10 μl of a 1 M sodium cyanoborohydride stock (in 10 mM NaOH) was added to each milliliter of the conjugation mixture, and the reaction mixture incubated at 37°C for 10 days with stirring. The conjugate reaction mixture was then dialyzed against distilled H_2_O using a 3,500-molecular weight cutoff Slide-A-Lyzer cassette (Pierce), sterilized with a syringe filter (0.45 µm), and lyophilized. A bicinchoninic acid (BCA) assay (Pierce) was used to quantitate the protein concentration of the glycoconjugate stock (and the remainder of the mass was assumed to be polysaccharide).

### Protein expression and purification.

Recombinant Hcp1 harboring an N-terminal 6× His tag was purified from E. coli TOP10(pBADBmhcp1-6HisF) as previously described (33). For expression of recombinant TssM with an N-terminal 6×His tag, the *tssM* open reading frame (BPSS1512) in which the Cys codon (TGC) at position 102 in rTssM was changed to a Gly codon (GGC) was cloned into pBAD/HisA (34). Briefly, plasmid pMB1000 (synthesized at GenScript, Piscataway, NJ) was digested with NcoI and HindIII to release the *tssM*-G102 insert, which was then cloned into similarly digested pBAD/HisA to produce pMB1001. Recombinant DNA techniques were conducted as previously described (49). DNA sequencing was performed by ACGT Inc. TssM was purified from E. coli TOP10(pMB1001) essentially as previously described for Hcp1 (33). The purities of Hcp1 and TssM were verified by SDS-PAGE. Protein concentrations were determined using a BCA protein assay kit (Pierce). Endotoxin removal was performed using high-capacity endotoxin removal resin (Pierce) per the manufacturer's instruction. The amounts of endotoxin in the Hcp1 and TssM preparations were quantitated by using an LAL chromogenic endotoxin quantitation kit (Pierce) per the manufacturer's instruction. Proteins were sterilized with a syringe filter (0.45 μm) and stored at 4°C.

### SDS-PAGE and Western immunoblotting.

Glycoconjugate samples were solubilized in 1× SDS-PAGE sample buffer and heated to 100°C for 5 min prior to electrophoresis on 4-to-20% Tris-HEPES gels (Pierce). Proteins were visualized via staining with Coomassie blue R-250. For Western immunoblotting, the glycoconjugate samples and controls were separated on the same 4-to-20% gels and electrophoretically transferred to nitrocellulose membranes. The membranes were blocked with 3% skim milk in high-salt Tris-buffered saline (HS-TBS; 20 mM Tris, 500 mM NaCl; pH 7.5) for 60 min at room temperature and then incubated for 1 h at room temperature with a 1/1,000 dilution of a B. pseudomallei CPS-specific MAb (3C5) (57). To facilitate detection, the membranes were incubated for 1 h at room temperature with 1/5,000 dilutions of an anti-mouse IgG-horseradish peroxidase conjugate (Southern Biotech). Blots were visualized using the Pierce ECL Western blotting substrate (Thermo Scientific) and a ChemiDoc XRS imaging system (Bio-Rad).

### Ethics statement.

All investigations involving animals were conducted in strict accordance with the recommendations in the *Guide for the Care and Use of Laboratory Animals* of the National Research Council (58). Protocols were approved by the Animal Care and Use Committees at the University of South Alabama (protocol 488113) or the University of Texas Medical Branch (protocol 0503014D). Mice were housed in microisolator cages under pathogen-free conditions, provided with rodent feed and water *ad libitum*, and maintained on a 12-h light cycle.

### Mouse immunizations.

Groups of 6- to 8-week-old female C57BL/6 mice (*n* = 16 per group; Charles River) were immunized subcutaneously on days 0, 21, and 35 with the following antigens: CPS-CRM197 (2.5 μg/dose of CPS as a conjugate), Hcp1 (5 μg/dose), TssM (5 μg/dose), CPS-CRM197 plus Hcp1, or CPS-CRM197 plus TssM. All antigens were formulated in tissue culture-grade PBS (pH 7.2; Gibco) with Alhydrogel 2% (500-μg/dose; Brenntag) and CpG (20-μg/dose; ODN 2006; Invivogen) as the adjuvant system. Mice immunized with adjuvant only served as controls.

### Analysis of antibody titers.

Terminal bleeds (*n* = 6 mice per group) were conducted 1 week after the final boost. Serum was stored at −80°C until required for use. Antibody responses directed against the vaccine antigens were assessed by ELISAs essentially as previously described (30, 56). Briefly, 96-well Maxisorp plates (Nunc) were coated overnight at 4°C with purified CPS, Hcp1, or TssM (1 μg/ml) solubilized in carbonate buffer (pH 9.6). The plates were blocked at room temperature for 30 min with StartingBlock T20 (TBS) blocking buffer (Pierce) and then incubated for 2 h at 37°C with the mouse serum samples serially diluted in Tris-buffered saline plus 0.05% Tween 20 (TBS-T; pH 7.5) plus 10% StartingBlock T20. To facilitate detection, the plates were incubated for 1 h at 37°C with 1/2,000 dilutions of anti-mouse IgM, IgG, IgG1, or IgG2b horseradish peroxidase-conjugated antibodies (Southern Biotech). The plates were developed with tetramethylbenzidine substrate (KPL) and read at 620 nm by using a FLUOstar Omega microplate reader (BMG Labtech). The reciprocals of the highest dilutions exhibiting optical densities that were 3 times the background levels were used to determine the endpoint titers for individual mice.

### Opsonophagocytosis assays.

The murine macrophage cell line RAW 264.7 (ATCC TIB-71) was maintained in Dulbecco's modified Eagle's medium supplemented with 10% (vol/vol) HI fetal bovine serum (DMEM-10; Invitrogen) and a standard mixture of antibiotics (100 U/ml penicillin, 100 μg/ml streptomycin, and 250 μg/ml amphotericin B; Sigma) at 37°C under an atmosphere of 5% CO_2_. Opsonophagocytosis assays were performed essentially as previously described (30). Briefly, RAW 264.7 cells resuspended in DMEM-10 were transferred into 24-well tissue culture plates at a density of 1 × 10^6^ cells/well and incubated overnight. B. pseudomallei K96243 cultures grown to early log phase were pelleted, resuspended at a density of 1 × 10^6^ CFU/ml in DMEM or DMEM containing 1% adjuvant only, Hcp1, TssM, CPS-CRM197, CPS-CRM197 plus Hcp1, or CPS-CRM197 plus TssM mouse immune serum (pooled and HI for 30 min at 56°C) and then incubated at 37°C for 1 h. RAW 264.7 monolayers were washed twice with Hanks' balanced salts solution (HBSS; Invitrogen) prior to the addition of the opsonized bacterial suspensions. The monolayers were incubated with the bacteria for 1 h at 37°C under an atmosphere of 5% CO_2_ and then washed twice with HBSS to remove extracellular bacteria. Infected RAW 264.7 cells were incubated with fresh DMEM-10 containing 250 μg/ml kanamycin to suppress the growth of residual extracellular bacteria. At 3 h postinfection, the infected monolayers were washed twice with HBSS and lysed with 0.2% (vol/vol) Triton X-100 (Sigma), and serial dilutions of the lysates were plated onto LB agar plates and incubated at 37°C for 48 h. Plate counts were used to enumerate bacterial loads.

### ELISpot assays.

Spleens (from 4 mice per group) were harvested 1 week after the final boost from terminally bled mice. Single-cell suspensions were prepared by passing the organs through 70-μm cell strainers (Falcon) into RPMI 1640 (Gibco) supplemented with 10% HI fetal bovine serum and 1× penicillin/streptomycin (Gibco) (RPMI-10). Cells were pelleted by centrifugation (500 × *g*), resuspended in red blood cell lysis solution (Sigma), incubated at room temperature for 10 min, pelleted (500 × *g*), and then resuspended in RPMI-10 at a concentration of 5 × 10^6^ cells/ml. Mouse IFN-γ ELISpot kits (R&D Systems) were used per the manufacturer's instructions. Splenocytes stimulated with CPS-CRM197, Hcp1, TssM, or medium only were added to the plates at a concentration of 2.5 × 10^5^ cells/well and then incubated for 48 h at 37°C under an atmosphere of 5% CO_2_. The ELISpot plates were processed and developed per the manufacturer's instructions. Plates were imaged using an ImmunoSpot S1 analyzer (Cellular Technology Ltd.). IFN-γ-secreting T cells were quantitated using the ImmunoSpot v5.1 professional DC smart count software (Cellular Technology Ltd.).

### LD_50_ determinations.

Groups of 16- to 18-week-old female C57BL/6 mice (Charles River Laboratories) were housed in standard microisolator cages and were provided water and food *ad libitum*. Mice were acclimated to housing for at least 7 days prior to bacterial infection. The LD_50_ for B. pseudomallei K96243 was determined by exposing three groups of mice (*n* = 6 to 8 mice/group) to 67, 728, and 53,500 CFU, as previously described (59). Mice were monitored for survival over 35 days, and the LD_50_ was calculated using methods described by Reed and Muench (60).

### Mouse challenge studies.

Five weeks after the final boost (day 70), the remainder of the immunized mice (*n* = 10 per group) were challenged with B. pseudomallei K96243 at a nebulizer concentration of ∼4.65 × 10^7^ CFU/ml via aerosol, essentially as previously described (59). Briefly, three groups of 20 mice were exposed to aerosolized bacteria via a three-jet collision nebulizer for 15 min at a constant flow rate of 30 liters/min. During this automated aerosol exposure, animals were restrained in a Biaera plastic aerosol rodent exposure box housed within a class III biological safety cabinet in a biosafety level 3 suite, using an automated aerosol exposure system. Animals were placed inside nose-only exposure restraint cones (In-Tox Products LLC, Moriarty, NM). Nebulizers were filled with 10 ml of LB broth containing the appropriate concentration of bacteria. Doses presented (Dp) to each group of animals were determined by performing standard CFU counts on the samples collected from an all-glass impinger (SKC BioSampler; SKC Inc., Eighty-Four, PA) containing LB broth with 4% glycerol and approximately 20 μl of antifoam 204 (Sigma-Aldrich). The Dp was calculated using the following formula: Dp (CFU) = C_Aero_ (CFU per milliliter) × exposure time (in minutes) × minute volume (in milliliters); the minute volume = 2.1 (weight [in grams])^0.75^. Weights and survival of the challenged mice were monitored for 35 days. Humane endpoints were strictly observed via daily monitoring throughout the study. Mice in the three challenge groups received Dp of 1,590, 1,650, and 1,550 CFU, which correlated with 10.3, 10.7, and 10.1 LD_50_s, respectively.

### CFU enumeration and histological evaluation.

At 35 days postchallenge, surviving animals were euthanized and their lungs, livers, and spleens were collected for CFU enumeration and histopathology. Half of each organ was fixed in 10% normal buffered formalin, and the remaining half was weighed and homogenized using Covidien Precision tissue grinders (Fisher Scientific). Tissue homogenates were serially diluted in PBS, plated, and incubated for 48 h at 37°C. Colonies were counted and normalized to organ weight (in grams). For histopathological analysis, fixed tissues were embedded in paraffin and sectioned prior to staining with hematoxylin and eosin (H&E). Pathology scoring was performed as previously described (59).

### Statistical analysis.

All graphs were produced by using GraphPad Prism 7.03 (GraphPad Software Inc.). Opsonophagocytosis and ELISpot data were analyzed using a Mann-Whitney U test. Survival data were analyzed using a log rank (Mantel-Cox) test.

## Supplementary Material

Supplemental material
