# Supplementary material for: Development of Subunit Vaccines That Provide High-Level Protection and Sterilizing Immunity against Acute Inhalational Melioidosis
Source: Infect Immun. 2017 Dec 19;86(1):e00724-17. doi: 10.1128/IAI.00724-17 (PMC5736816; doi:10.1128/IAI.00724-17)
Supplement: Supplemental material [file supp_86_1_e00724-17__index.html]

Supplemental material 

# Development of Subunit Vaccines That Provide High-Level Protection and Sterilizing Immunity against Acute Inhalational Melioidosis

## Supplemental material

- Supplemental file 1 -

  Fig. S1. Western immunoblot analysis of CPS-CRM197. Fig. S2. SDS-PAGE analysis of recombinant *B. pseudomallei* Hcp1 and TssM antigens. Fig. S3. Determination of the inhalational LD50 of *B. pseudomallei* K96243 for C57BL/6 mice. Table S1. Histopathological analysis of mouse tissues following a lethal inhalational challenge with *B. pseudomallei*.

  PDF, 143K
