# Supplementary material for: Development of Subunit Vaccines That Provide High-Level Protection and Sterilizing Immunity against Acute Inhalational Melioidosis
Source: Infect Immun. 2017 Dec 19;86(1):e00724-17. doi: 10.1128/IAI.00724-17 (PMC5736816; doi:10.1128/IAI.00724-17)
Supplement: Supplemental material [file IAI.00724-17_zii999092270s1.pdf]

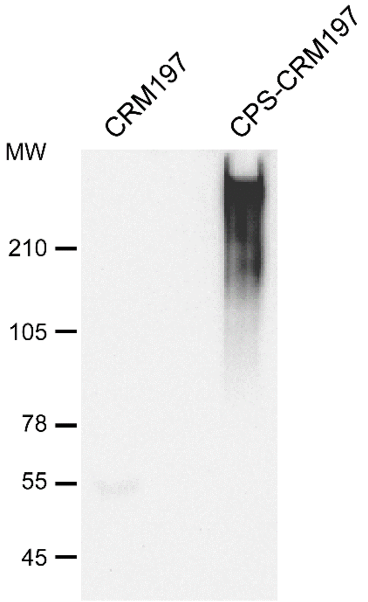

FIG S1 Western immunoblot analysis of CPS-CRM197. CRM197 (control) and CPS-CRM197 were separated on a 4-20% Tris-HEPES gel and electrophoretically transferred to a nitrocellulose membrane. CPS was detected using the *B. pseudomallei* CPS-specific mAb, 3C5 (see Materials and Methods). The positions of the protein molecular standards (kDa) are indicated on the left.

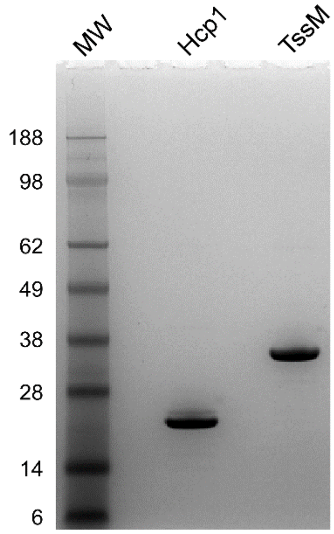

FIG S2 SDS-PAGE analysis of recombinant *B. pseudomallei* Hcp1 and TssM antigens. Hcp1 and TssM were separated on a 4-12% Bis-Tris Bolt gel and visualized with Coomassie Blue R-250 (see Materials and Methods). The protein molecular standards (kDa) are indicated on the left.

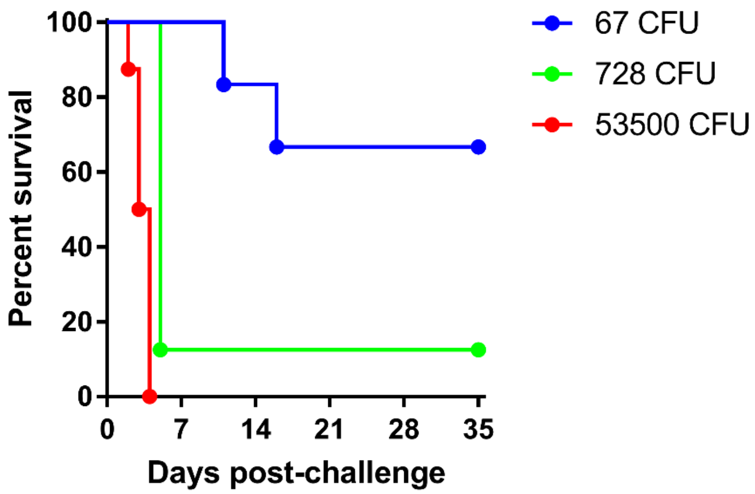

FIG S3 Determination of the inhalational LD_{50} of *B. pseudomallei* K96243 for C57BL/6 mice. Groups of 16-18 week-old, female C57BL/6 mice (n = 6 to 8 mice/group) were exposed to 67, 728 and 53,500 CFU of *B. pseudomallei* K96243 as previously described (see Materials and Methods). Mice were monitored for survival over 35 days and the LD_{50} calculated.

TABLE S1 Histopathological analysis of mouse tissues following a lethal inhalational challenge with *B. pseudomallei*.

Specimen Name	Grade*	Granulomas**	Lobar Pneumonia**
Mouse 1 - Lung	0	0	No
Mouse 1 - Liver	0	0	-
Mouse 1 - Spleen	0	0	-
Mouse 2 - Lung	0	0	No
Mouse 2 - Liver	0	0	-
Mouse 2 - Spleen	0	0	-
Mouse 3 - Lung	0	0	No
Mouse 3 - Liver	0	0	-
Mouse 3 - Spleen	0	0	-
Mouse 4 - Lung	0	0	No
Mouse 4 - Liver	0	0	-
Mouse 4 - Spleen	0	0	-
Mouse 5 - Lung	0	0	No
Mouse 5 - Liver	0	0	-
Mouse 5 - Spleen	0	0	-

* Histopathology Grading System: 0 = no lesions, 0% of the tissue is affected; 1 = minimal change, 10% and less of the tissue is affected; 2 = mild change, 10-25% of the tissue is affected; 3 = moderate change, 25-50% of the tissue is affected; 4 = marked change, 50-75% of the tissue is affected; 5 = severe change, greater than 75% of the tissue is affected.

** Additional features evaluated: Number of large granulomas (>500 microns); Lobar pneumonia (Yes/No).
